# A three-step process of Nphp3 ciliary localization

**DOI:** 10.1186/2046-2530-1-S1-P46

**Published:** 2012-11-16

**Authors:** D Shiba, K Nakata, H Fukui, D Kobayashi, T Yokoyama

**Affiliations:** 1 Kyoto Prefectural University of Medicine, Japan

## 

Primary cilia are microtubule-based organelles projecting from the surface of nearly all cells. Primary cilia are complex organelles and are structurally divided longitudinally into sub-compartments that include the basal body, the transitional zone, the ciliary shaft and the tip. Nephronophthisis (NPHP) is an autosomal recessive cystic kidney disease with 11 responsible genes (*NPHP1-11*) thus far being identified. The causative gene products, nephrocystins, are divided into at least two groups based on these localizations. Group I nephrocystins (Nphp1, 4, 5, 6 and 8) are localized in the transitional zone, whereas group II nephrocystins are localized in the Inv compartment (Nphp2, 3 and 9). Here, we show the localization mechanism of Nphp3. We generated a series of GFP-tagged deletion constructs of Nphp3 and tried to find the ciliary targeting sequences of Nphp3. We found that the N-terminal fragments, Nphp3 (8–201), Nphp3 (52–201) and Nphp3 (96–201), that contain the CC domains, targeted the basal body, but could not enter into the ciliary shaft. Further analysis revealed that an N-terminal glycine (G2), which is a conserved myristoylation site among vertebrates, is also essential for trafficking of Nphp3 to the ciliary shaft. We revealed that Inv/Nphp2 is not required for entry of Nphp3 into the ciliary shaft. Following entry of Nphp3 into the ciliary shaft, Inv/Nphp2 is required for the localization of Nphp3 to “the Inv compartment”. Our results showed the importance of myristoylation in ciliary trafficking, and suggest that Nphp3 ciliary localization occurs in a three-step process.

http://www.f.kpu-m.ac.jp/k/anat2/English/index.html

